# A Food System Approach for Sustainable Food-Based Dietary Guidelines: An Exploratory Scenario Study on Dutch Animal Food Products

**DOI:** 10.3389/fnut.2021.712970

**Published:** 2021-09-01

**Authors:** Corné van Dooren, Laila Man, Marije Seves, Sander Biesbroek

**Affiliations:** ^1^Netherlands Nutrition Centre (Voedingscentrum), The Hague, Netherlands; ^2^Division of Human Nutrition and Health, Wageningen University, Wageningen, Netherlands

**Keywords:** food system scenarios, sustainable food-based dietary guidelines, animal food products, environmental impact, healthy diets, livestock size

## Abstract

This study explores interconnections between food consumption and production of animal (by-)products in different food system scenarios within the scope of Dutch Food-Based Dietary Guidelines (FBDG). For this scenario study, a Microsoft Excel model was created that include seven scenarios with different quantities of eggs, milk, cheese, beef cattle, broilers, and pigs as input. Number of animals, intake of energy, animal protein, saturated fatty acids (SFAs), trans-fatty acids (TFAs), salt, greenhouse gas emissions (GHGEs), and land use (LU) were calculated and compared with current consumption and reference values. Based on the concept of eating the whole animal, every recommended lean, unprocessed portion of beef comes along with a non-recommended portion of beef (two portions for pork, 0.5 portion for broilers). The reference values for SFAs, TFAs, and salt were not exceeded if the intake of meat is limited to 410 g/week. The scenarios with recommended 450 mL semi-skimmed milk and 40 g/day low-fat cheese results in 36 g/day of butter as by-product, exceeding its acceptable intake three times. The near-vegetarian scenario with recommended amounts of eggs, milk, and cheese, includes only a portion of beef/calf per 6 days and a portion of chicken per 9 weeks as by-products. This scenario more than halves the GHGE and LU. Finally, the scenario that included the maximum recommended amounts of animal products is reachable with half the current size of Dutch livestock. This conceptual framework may be useful in the discussion on how future sustainable FBDG can incorporate a more food system-based approach.

## Introduction

### FBDG Should Be Sustainable

Worldwide, governments have agreed to prioritise actions toward sustainable development in line with the United Nations Sustainable Development Goals (1). Food systems are highly relevant for this sustainable development (2, 3). It is widely acknowledged that current food systems are not ecologically sustainable. Food production and consumption are not within the so-called safe operating space, thereby not complying to ecological nor health objectives (4). In fact, food production and consumption are two of the main drivers of global climate change (3, 5). Concerns about ecological boundaries will only increase further if no steps are taken toward more sustainable food systems globally (6). Although there has been increased focus on this topic in recent years, many gaps in the knowledge of the relationship among environmental factors, food systems, and nutritional outcomes persist (7).

### Current Diets and Recommendations

At present, global food production and consumption have a share of 21–37% in total greenhouse gas emissions (GHGEs) and up to 40% in land use (LU) (8). The same is true for the Dutch consumption (9). Animal (-derived) food products such as red and white meat, milk products, and cheese are the largest contributors to the ecological impact of the current diet (10). In the Netherlands, animal (-derived) foods together contribute to about 60% of the total diet-related GHGEs. However, the present calculations concerning ecological impact underlying the Food-Based Dietary Guidelines (FBDG) are based on individual food products and their consumption rather than interrelated products within a closed food system [e.g., (11)].

The Federation of European Nutrition Sciences established a task force for developing a conceptual framework for the future development of FBDG in Europe. One of the conclusions was that environmental aspects should be included in the future conceptual framework for FBDG (12). In addition, a further study needs to be done exploring current practises, existing methodologies, and the prospects for incorporating other relevant dimensions into a future conceptual framework for Sustainable FBDG in Europe (12). Also, the Food and Agriculture Organisation (FAO) and WHO embrace the concept of sustainable dietary guidelines. They developed guiding principles around what constitutes sustainable healthy diets, to be further translated into clear, non-technical information and messages to be used by governments and other actors in policy-making and communications (13). Several European countries have already taken some dimensions of sustainability into account in their most recent FBDG (14), which include some ecological perspectives in The Netherlands (15). Nevertheless, only a slight reduction in the dietary impact of the Dutch diet on GHGE and LU will be achieved if the current maximum amount of recommended meat (500 g/week) would be consumed (11). The GHGE of the Dutch guidelines are relatively high compared with those of other countries (16), so there is a need for further development of FBDG in the direction of sustainability.

### Dependencies in the Food System

A knowledge gap exists on the ecological impact of Dutch FBDG from a food system perspective. Typically, research on how to achieve more sustainable diets has focussed on two ways. First, the production pathway in which reducing the ecological footprint of animal (-derived) products per kg of product is emphasised [e.g., use of feed from waste streams (17)]. Second, the consumption pathway, which focuses on eating less or no animal (-derived) products, for instance, switching to a vegetarian or vegan diet (18). However, neither of these approaches consider the elements as operating in one food system (19). The analysis of Springmann et al. (20) suggests that national guidelines could be both healthier and more sustainable by providing clearer advice on limiting, in most contexts, the consumption of animal source foods, in particular beef and dairy. Therefore, this study focuses on animal food products.

Questions that arise when adopting a food system approach are, for instance: could the ecological impact of the FBDG be reduced by consuming roosters (vs. only broilers) as a source of chicken meat, or by consuming meat from laying hens and/or dairy cows and their calves? Could the ecological impact of the FBDG be reduced by consuming all parts of the animal instead of only lean cuts? And what would be the nutritional consequences? Despite the substantial evidence showing the need and possibilities for aligning health and environmental objectives, only a minority of countries have, so far, included environmental sustainability in their FBDG, but none with a food system approach (21). In fact, the majority of research on sustainable diets tends to focus on individual food products rather than products within a food system (22). The focus of this article is an exploratory scenario study on Dutch animal food products in relation to the Dutch FBDG. The Dutch food system and guidelines are comparable with those of several other northern European countries such as Belgium, Germany, United Kingdom, and Nordic countries. Therefore, the objective of this study is to explore different food system scenarios, ultimately to investigate the implications of the results on future sustainable FBDG in the Netherlands. Thereby, the focus will be on a closed loop of food grade (safe for human consumption) animal (by-) products between production and consumption on a national level.

## Methods

For this investigation, we conducted an exploratory scenario study on Dutch animal food products and interdependencies in the animal food system using a model built in Microsoft Excel.

### Data Collection

A data set in Excel was created that integrates both extensive information on the livestock and its by-products and interdependencies, and information on the production and consumption of livestock production systems in the Netherlands. The studied livestock systems are laying hens and broilers, dairy and beef cattle, veal cattle, and pigs.

For each livestock production system, the model includes:

- The animal components (see Section Building the Model) of the system and their interdependencies, such as portion weight and ratios between different components of each livestock production system
- The nutritional value (energy, animal protein, SFA, TFA, salt) of each component, and the separation in edible and marketable meat and dairy parts
- The current nutritional advice regarding these components according to the Dutch FBDG
- Categorisation of the components in or outside the Wheel of Five (classification is explained in Section Nutritional Classification of Animal Products)
- The ecological impact (GHGE and LU) of each component, including processing and transport
- Consumption and production statistics of each component.

For nutritional data of the components, the Dutch Branded Food Database was used (23). Regarding production data, mainly the open data source StatLine from Statistics Netherlands CBS was used (24, 25). An exception was production data of dairy: these were extracted from ZuivelNL, because they were more detailed (26). Consumption data were extracted from the Dutch National Food Consumption Survey 2012–2016 (27). The LCA database of the RIVM was used (28), such as the extrapolated data for the RIVM healthy diets study (11). The GHGE and LU data are generated by life cycle analysis according to the Agro-Footprint method (29). Missing data were taken from data sources by Blonk Consultants (30). We selected GHGE and LU as they cover most of the diet-related ecological impact. Although nitrogen emission through manure and ammonia is an important ecological impact in the livestock production system, we lack data on the nitrogen footprint per product (31).

Data required to calculate ratios between the components of the livestock production systems (productivity, yield, edible weight) were taken from different sources:

- productivity overall: Blonk Consultants or Blonk Agri-footprint BV (26, 29, 32),
- dairy system: ZuivelNL (26) (dairy producer platform),
- chicken system: Plukon Food Group, Kipster, www.kipinnederland.nl (association of Dutch poultry producing firms) and
- beef and pork system: SVH, www.vlees.nl (meat producer platform).

The quantities of edible weight per animal are based on Luske and Blonk (32). The sources were selected after consultation with three experts in this field.

### Nutritional Classification of Animal Products

**Figure 1:**
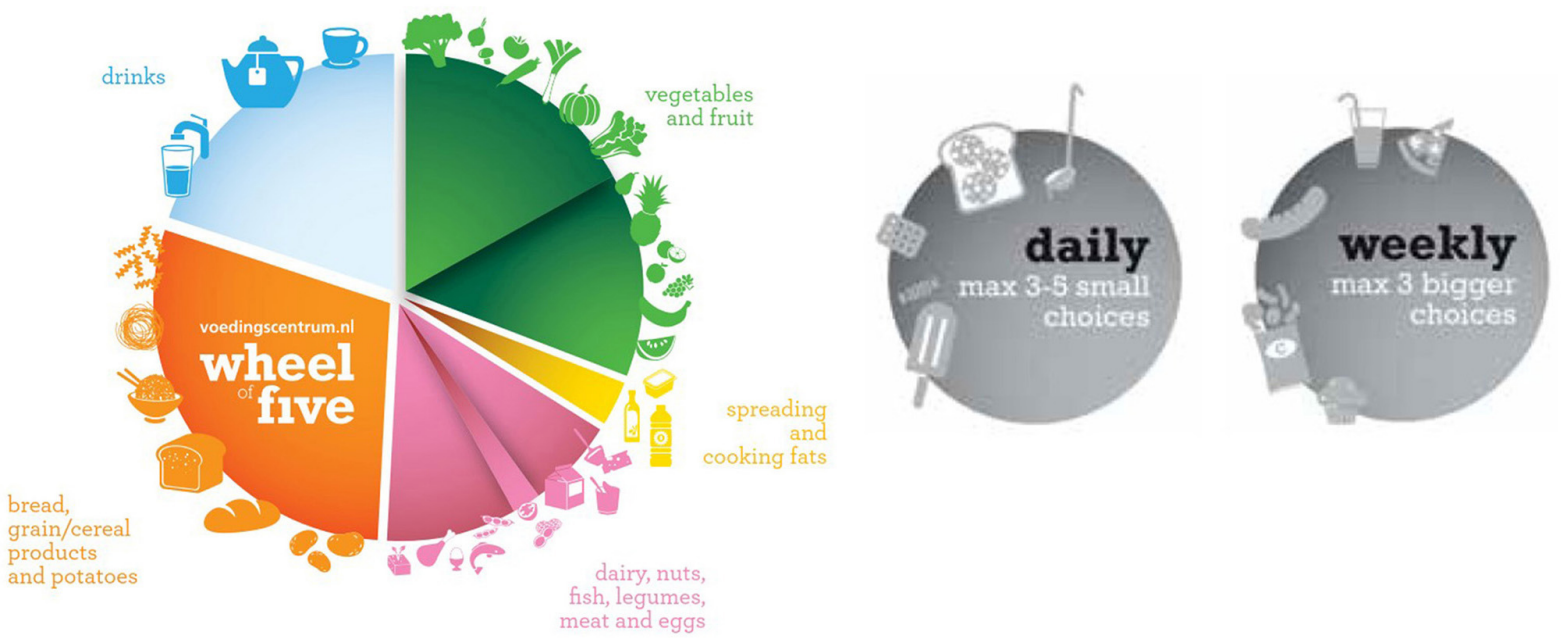
Wheel of Five with the five food groups and a visualisation of Daily and Weekly choices (33).

Currently, the Dutch FBDG are set up in the form of a nutritional education model: The Wheel of Five, further referred to as “the Wheel” [see Figure 1; the five groups; bread, grains, and potatoes; drinks; fruits and vegetables; fats; and dairy, nuts, fish, legumes, meat, and eggs; (33)]. The Wheel models an optimal combination of food product groups that both maximises health benefits and satisfies nutritional needs according to the recommendations of the Health Council of the Netherlands (34, 35). The general advice is to eat especially food products included in the Wheel (divided over five main categories) and to limit the consumption of products that fall outside the Wheel (divided in the categories “Daily choice” and “Weekly choice”), explained in more detail elsewhere (21). One of the main recommendations of the Health Council of the Netherlands and the Wheel is to eat less animal-based and more plant-based products (35). In this study, several limitations are set. From a health perspective, the consumption of red and processed meat is limited to a maximum of 300 g/week; from a sustainability perspective, the consumption of total meat is limited to a maximum of 500 g/week, below the current consumption. Besides limitations in product groups, there are limitations set for health reasons on SFA, TFA, and salt intake. A food product that is included in the Wheel needs to adhere to certain criteria, which are different for each of the categories of the Wheel. The FBDG has placed those products that are not included in the Wheel of Five into two categories: Daily choices and Weekly choices (Figure 1). The basic principle, when drawing up criteria for Daily choices, is that it should be possible to select foods from outside the Wheel of Five several times a day (33). Food products that fall outside of the Wheel and contain ≤ 75 kcal, ≤ 1.7 g SFA, and ≤ 200 mg Na (0.5 g salt) per portion are Daily choices. Otherwise, they are called Weekly choices. For adults, it is advised to limit the consumption of Daily choices to no more than three to five times a day, while the consumption of Weekly choices should be limited to no more than three times per week, limiting average energy intake from foods outside the Wheel to no more than 15% of total energy intake (21, 36). These criteria result in the major part of the current meat, dairy, and cheese consumption not being included in the Wheel.

### Building the Model

#### Connecting Consumed Meat Products to Primal Meat Cuts

Judge et al. (37) was used to determine the primal cuts of beef cattle (similar data for dairy cattle were unavailable) and pork. Judge et al. (37) used data from a pre-existing data source from the Irish Cattle Breeding Federation, describing Irish beef cattle. These data were considered applicable to the Dutch beef cattle system, as many luxury beef products (i.e., prime cuts, unprocessed beef) in Dutch supermarkets originate from Irish beef. Moreover, many of the breeds mentioned in the study are known to be used as Dutch beef cattle: Charolais, Belgian Blue, and Limousin (37). Websites of Dutch butchers and chefs were used to fill the information gap (i.e., which beef and pork products originate from which primal cuts). Pork Checkoff (38) was used to determine the primal cuts of pork. The cuts of chickens were received from the experts at Plukon Food Group and Kipster.

#### Connecting Meat Consumption to Animal By-Products: Consumable Organs

Animal by-products are products that livestock produce that are not intended or used for human consumption in the Netherlands. Intended in this sense does not necessarily imply that the by-products are not suitable for human consumption. For instance, some organs are suitable for human consumption (e.g., not harmful to human health when consumed, for example liver and kidney); however, there is no market for these organs in the Netherlands. Other by-products are not suitable for human consumption and are instead used as feed, pet food, or biochemicals, or burned because of harmful contents (32). The cuts of carcasses of the six selected livestock animals are categorised in five components in the model: Wheel (meat, excluding organs), organs in the Wheel, Daily choices, Weekly choices, and unknown (inedible/not marketed) (Table 1).

**Table 1:** Percentage of components within each livestock production system based on weight.

**Livestock**	**Hot carcass weight (kg)**	Edible weight^*^ (kg)	**Wheel (meat, excluding organs) (%)**	**Organs in the Wheel (liver, kidney) (%)**	***Wheel, total (%)***	**Daily choices (%)**	**Weekly choice (%)**	***Non-wheel (known) (%)***	**Unknown (i.e., inedible/not marketed, including bones) (%)**
Dairy cattle	307	151	38	1	39	6	25	31	29
Calves	160	79	44	15	59	00	25	25	15
Beef cattle	464	212	38	1	39	6	25	31	29
Laying hens	2.15	1.35	0	0	0	0	63	63	37
Roosters	0.90	0.80	48	0	48	0	41	41	11
Broilers	1.65	1.03	44	8	52	1	9	11	38
Pigs	110	77	22	1	23	17	29	46	31

*Numbers are depicted in % of edible weight unless indicated differently*.

* *Edible weight: excludes head, skin, and internal organs. Includes bones, fat, and moisture*.

#### Connecting Dairy Consumption to Dairy Herd Meat and Dairy Fats Consumption

**Figure 2:**
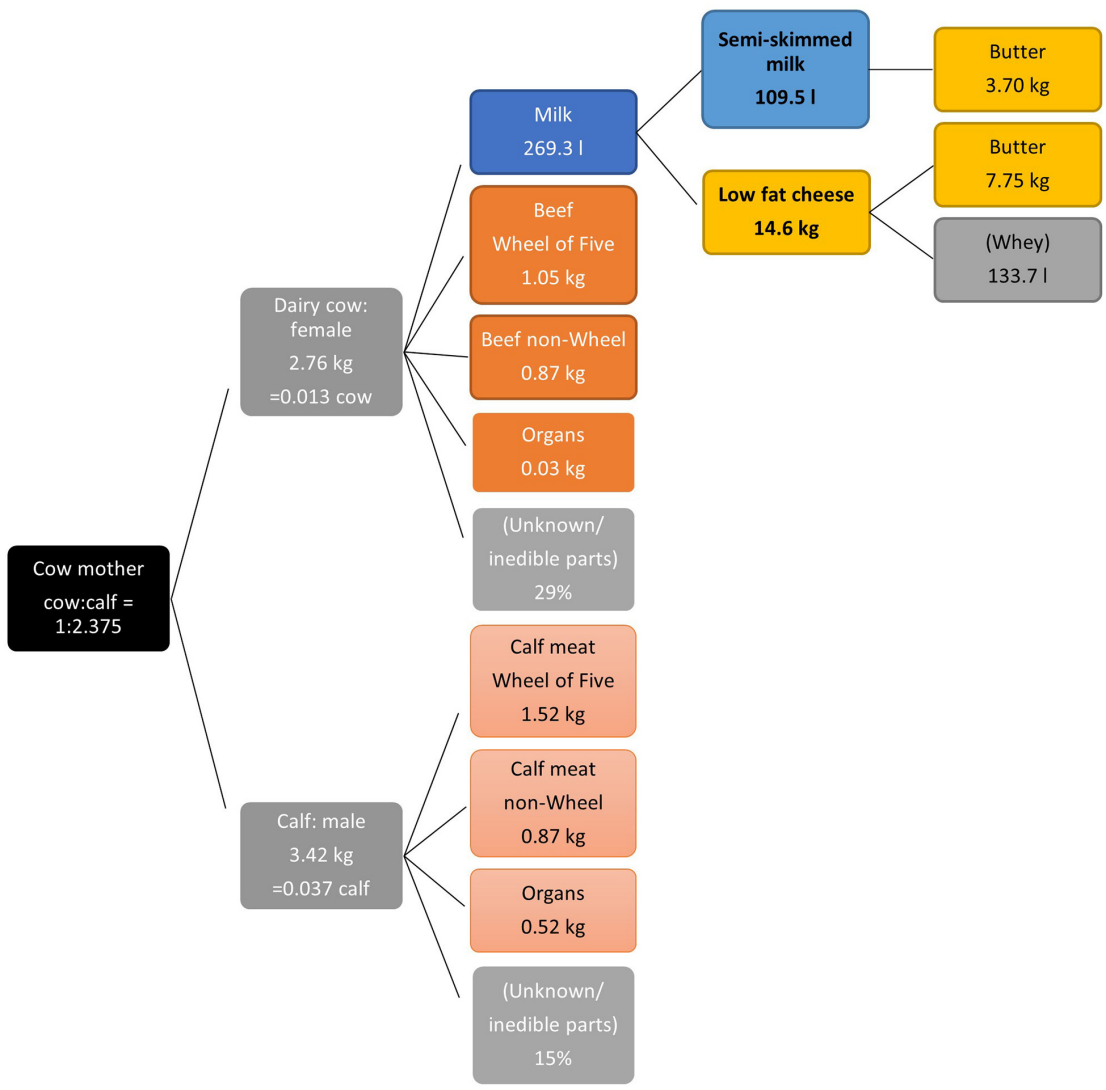
Model with interdependencies in the dairy cow system (products in parenthesis are outside the model), illustrated by the quantities related to the recommended portions of semi-skimmed milk and low-fat cheese per year for adults 19–50 years old.

The dairy cow system is more complicated than the other livestock systems (Figure 2); therefore, some assumptions were made. A Dutch dairy cow delivers, on average 4.75 calves in her lifespan, so the ratio dairy cow:calf is 1:4.75. It was assumed that half of the calves will be available for their meat (i.e., the male ones, ratio 1:2.375), and that the other female half will be fully used for replacement of the dairy herd. In fact, this percentage is somewhat higher, as an unknown minor part of the female calves is also available for their meat (39). Based on slaughter weight, the model calculates the quantity of edible meat per animal. Figure 2 shows an example of the quantities related to the recommended portions of semi-skimmed milk and low-fat cheese per year for adults 19–50 years old: 109.5 L milk and 14.6 kg cheese.

Dutch milk production per cow is calculated as on average 30,000 L per life span (39). A litre of semi-skimmed milk (1.5% fat) has a by-product of 33.8 g of butter, because cow milk has an average fat percentage of 4.1%. Per 1 kg of cheese, about 10 L of milk is needed on average. One kg of low-fat cheese (30% fat in dry weight) requires 10.69 L of milk and yields a by-product of 530.9 g of butter and 9.16 L of whey [based on (26)]. For the example in Figure 2, this means 3.7 kg butter from milk and 7.75 kg butter from cheese production per year, derived from 269.3 L of milk from the cow. The amount of whey produced as a by-product of cheese was depicted in the model to give an indication of its volume. Fat as by-product from dairy and cheese was modelled as butter. Fat could be processed into cream as well, which would yield a different amount of product. Whey is applied for human consumption as an ingredient for soft drinks (Weekly choice) or as baby formula. However, further analysis on whey was beyond the scope of this study. Based on the ratio of 30,000 l milk:dairy cow, this amount of milk is equivalent to 2.76 kg of cow. Based on the ratio cow:calf, 2.76 kg per cow (= 0.013 cow) is equivalent to 3.42 kg calf (= 0.037 calf). The 2.76 kg beef and 3.42 kg of calf are divided in several edible and unknown/inedible parts based on the distribution given in Table 1.

#### Connecting Egg Consumption to Hen and Rooster Consumption

Egg production is an agricultural sector separate from chicken meat production. Within the egg production sector, hens are slaughtered after productive life and marketed most of the time in their entirety as chicken for soup (Weekly choice). The assumption is that in the system for every hen there is one rooster, but with a lower average slaughter weight: 0.9 vs. 2.15 kg. The lifetime egg production per chicken is on average 383 eggs (29). Per 100 g egg (two pieces of 50 g), the by-product is 7.03 g of hen meat and 4.17 g of edible rooster meat. Roosters are normally killed after birth and used as feed for pets or zoo animals. An exception is a commercial farmer in The Netherlands that raises roosters to be sold as chicken meat (www.kipster.nl). The assumption is that this business model can be upscaled to the whole sector.

#### Connecting Pork Consumption to Feeding Pork Solely on Residue Streams

Ruminants such as cows can be solely fed on grass, but not monogastric animals such as pork. According to Elferink et al. (17), the Dutch food industry has four main residue streams that are used as pork feed. The potato industry has a residue stream of peals (23%), which has a quantity of 7 kg per capita per year. The sugar industry has two residue streams from sugar beets: dried beet pulp (24%) and molasses (17%), accounting for 18 and 12 kg per capita per year. Based on these inputs, an amount of pork can be raised equal to 28 g per capita per day (196 g/week). The fourth residue stream is soybean scraps, but we interpret this as a regular input stream rather than a residue stream, because it involves 80% of the soybean. This stream accounts for 72 kg of pork per capita per year, which is equal to 53 g/day (17). Chicken can also be fed on residue streams, but we have no access to data calculating the conversion form residues to chicken meat.

#### Connecting Food System Production to Recommended Quantities of Animal Products in Dutch FBDG

For classification of the components of the livestock production systems into Wheel product, Daily choice, or Weekly choice, the criteria from Brink et al. (21) were used (see Section Nutritional Classification of Animal Products.). Males (19–30 years old) are used as reference, because it is the population group with the highest overall consumption, consumption of meat, and environmental impact of their diet (10). Table 2 shows the current consumption of this group per week, compared with the recommended quantities.

**Table 2:** Recommended intake of animal products (g/mL) for adults per week in The Netherlands (36) and the current consumption of males 19–30 years old (27) in g/week.

	**Minimum recommendation (g/week)**	**Maximum recommendation (g/week)**	**Current consumption (g/week) (males 19–30 y)**
Total meat	0	500	864
Red meat (beef, pork)	0	300	683
White meat (chicken)	0	500 minus red meat	181
Milk products (milk, yoghurt)	2,100	3,150	2,389
Cheese (low fat 40 g/day)^a^	210	280	258
Butter (preference: soft liquid oils)^b^	0	84 semi-skimmed	8.2
Spreadable and cooking fats	455	–	165
Eggs (2–3)	100	150	85
Fish (1 portion)^*^	100	–	109

a *For mathematical reasons, a lower limit for cheese was required for the model; thus, the lower limit for cheese was set at 30 g a week, which is in line with previous Dutch FBDG*.

b *1 portion butter is 6 g, 44 kcal, 3.2 g saturated fat and <0.1 g salt (Weekly choice), semi-skimmed butter is 20 kcal, 1.5 g saturated fat, 24 portions butter = 1,050 kcal, 143 g/week*.

* *Fish is not included in this study*.

Regarding animal meat, it is advised to limit the consumption to a maximum of 500 g a week, of which a maximum of 300 g for red meat. The advice for dairy (e.g., semi-skimmed milk, yoghurt) is to consume two to three portions (150 mL) per day; for low fat cheese, there is a recommendation of 40 g per day. Furthermore, it is advised to consume two to three eggs à 50 g per week. Finally, the advice for spreadable and cooking fats is 65 g per day for males in the age of 19–30 years old (36). The model allows a maximum of two Daily choices per day (14 per week; = <1,050 kcal) and 1 Weekly choice per week for animal-derived products (set as a portion of 100 g meat) (allowing room for other choices such as alcoholic drinks, soft drinks, snacks, cakes, and sweets).

### Model Scenarios

The model makes it possible to run a range of scenarios, but for this study seven possible scenarios were selected to evaluate the consequences of connecting recommended intakes (FBDG) to consuming all interrelated animal products: first, two scenarios to explore the impacts of the maximum nutritional recommendations (1a, 1b); second, two scenarios to explore the minimum nutritional recommendations (2a, 2b); third, two scenarios to explore the maximal utilisation of existing waste streams (3) and existing livestock (4). The scenarios are:

- Scenario 1a: current FBDG with maximum of 500 g meat, of which 300 grammes is for red meat in beef (maximum impact scenario)
- Scenario 1b as 1a: 300 g red meat of which 188 grammes is of pork and 112 g beef (realistic scenario)
- Scenario 2a: FBDG only including chicken from laying hens and beef from dairy cattle and calves (minimum impact, near-vegetarian scenario)
- Scenario 2b: FBDG as 2a near vegetarian, including chicken roosters (minimum impact, rooster scenario)
- Scenario 3: FBDG as 2b including pork raised on residue streams of the food industry (28 g pork per day) and including current beef cattle, but only grass-fed or grazing in nature sites (food system optimal scenario)
- Scenario 4: FBDG as 3 with maximal utilisation of existing Dutch livestock (broilers, beef cattle) up to 500 g meat (agriculturally optimal scenario).

The quantities of meat mentioned in scenarios 1a, 1b, 3, and 4 are the input quantities for the model. Per scenario, the environmental impact on intake of energy and five nutrients, limits in consumption related to the maximum of 2 Daily and 1 Weekly choices, and effects on the livestock size were evaluated (assuming the whole population eats according to the scenario). Based on the results of Scenarios 1-4, Scenario 5 (nutritionally optimal scenario) was calculated at the end, looking at maximal utilisation of livestock within the nutritional recommendations, i.e., keeping the Daily and Weekly choices within the limits of FBDG and including all livestock systems in a more optimal amount (300 g white and a maximum of 200 g red meat).

## Results

### Consumption of Animal Products per Scenario

Table 3 gives an overview of the consumption of animal products per scenario in grammes per week. The inputs for the model were the quantities of eggs, milk, and cheese, set as constraints for the scenario. The outputs of the model were quantities of animal meat (laying hens, veal, dairy cattle, etc.).

**Table 3:** Consumption of animal products per scenario in g/week.

**Scenario**	**Eggs**	**Dairy**		**White meat**			**Red meat**					**Total meat**
		**Cheese**	**Milk**	**Broilers**	**Roosters**	**Laying hens**	**Pork**	**Beef cattle**	**Veal**	**Dairy cattle**	**Total red meat**	
1a	150	280	3,150	180	0	11	0	191	66	44	300	491
1b	150	280	3,150	189	0	11	188	0	67	45	300	500
2a	100	210	2,100	0	0	7	0	0	46	31	77	84
2b	100	210	2,100	0	4	7	0	0	46	31	77	89
3	100	210	2,100	0	4	7	196	17	48	32	292	304
4	150	280	3,150	183	6	11	171	17	67	45	300	500
5	150	125	2,100	283	6	11	40	17	38	25	120	420

### Part of Animal Consumption Within the Wheel of Five

Table 1 demonstrates that different animals have a different yield in total Wheel products, ranging from 0 (laying hens consumed in whole such as in soup) to 52% for broilers and 59% for calves of the hot carcass weight (i.e., the carcass excluding the head, skin, and internal organs and including bones, fat, and moisture). This is related to the percentage of available lean and fresh, unprocessed cuts. Based on the available data, organ consumption has high potential for broilers (13%) and beef (calves: 15%). Broilers and calves fit best into the Wheel, and laying hens do not fit at all, because they are sold as one piece. Pigs are the livestock with the lowest part lean, unsalted, unprocessed meat of 22%. This means that in practise of a food system approach, which includes eating the animal from nose to tail, along with every portion of beef in the Wheel, a portion of beef Weekly choice is expected to be eaten. For pork, along with every portion in the Wheel, two portions of pork Weekly choice are expected to be consumed, and for broilers, along with every portion in the Wheel, 0.5 portions of broiler Weekly choice.

### Effect on Intake of Saturated Fats and Salt

Reduction in the consumption of meat will result in changes in nutrient intake. Considering not only recommended meat cuts in the model but also Daily and Weekly choices result in an increased consumption of saturated fatty acids (SFAs), trans-fatty acids (TFAs), and salt. Moreover, compared with the current diet, the daily intake of SFA and TFA is higher in all maximum scenarios (i.e., upper limits of FBDG, see Table 4), but lower in all minimum scenarios (i.e., lower limits of FBDG, scenarios 2a, 2b, and 3). Only scenarios 1a, 1b, and 4 exceed the current consumption of SFA and TFA in the reference diet and the reference value for SFAs. These scenarios also contain more than 40% protein from animal sources. Salt intake decreases in all the scenarios and is below the reference value of 2.17 g. Scenario 5 demonstrates the best results for SFAs, TFAs, and salt (and calories) and still provides a substantial portion of animal protein (30.8 g/day).

**Table 4:** Nutritional intake per day by consumption of animal (-derived) products of differing scenarios and of reference (=current) diet.

**Scenario**	**Energy (kcal/day)**	**Animal protein (g/day)**	**SFA (g/day)**	**TFA (g/day)**	**Salt (g/day)**
Reference values (males 19–30 y)		36.4 (40%)	29.5 (10 en%)	3.0 (1 en%)	6.00 (2.17 from animal products)
Reference diet	885	*58.6*	23.7	0.66	*2.82*
1a	712	*44.8*	*30.3*	0.76	1.55
1b	733	*44.5*	*31.4*	0.72	1.84
2a	444	23.7	20.5	0.49	0.94
2b	444	23.8	20.5	0.49	0.94
3	511	29.6	22.6	0.52	1.39
4	723	*44.6*	*31.4*	0.73	1.81
5	451	30.8	17.5	0.40	1.00

*In italics the exceeded intakes compared to the reference values. Reference values for males 19–30 years old are based on a 2,645-kcal diet, 36% salt intake from meat and dairy, and reduction of animal-based protein intake to 40% (27, 36)*.

Additionally, the consumption of dairy and eggs is inevitably linked to some beef meat (dairy cow and calf) and chicken meat (laying hen and rooster). More specifically, more beef meat is available than chicken meat regardless of the (lower or upper) limits of the FBDG. Consumption of dairy and eggs following scenario 2a, in case no extra streams of meat exist (no beef cattle nor broilers) and in case no roosters are consumed (like in the current situation), 77 g of beef meat per week (ca. 1 portion of 100 g each 9 days) vs. 7 g of chicken meat per week (ca. 1 portion each 14 weeks) is available. Thus, when following the FBDG on dairy and eggs in the current situation, consumption of about 1 portion of beef (dairy cow and calf) each 6 days is still within the borders of the food system, whereas the consumption of chicken (laying hen) is much less frequently allowed by the food system: one portion each 9 weeks (scenario 2b).

### Results on GHGE and LU

Table 5 summarises the GHGEs and LU per scenario, compared with the planetary boundaries. According to Wood et al., the boundaries for food per person are respectively, 0.89 kg CO_2_-eq/day and 2.68 m^2^*year/day (4). Concerning GHGE, consumption of animal food products according to the FBDG in the current situation (i.e., in a food system without roosters and little veal but with beef cattle and broilers) leads to GHGE of 3.34 kg CO_2_-eq/day (Scenario 1a). This diet, following the upper limits of FBDG, still leads to lower GHGE than the reference diet (3.53 kg CO_2_-eq/day), but also exceeds the planetary GHGE boundary per day. Scenario 2a (minimum impact, near-vegetarian scenario) demonstrates the lowest climate impact (1.46 kg, −59%) but still exceeds the planetary boundary. Regarding LU, consumption of animal food products according to the FBDG in the maximum impact scenario leads to an LU of 2.44 m^2^*year/day (scenario 1a), which is more than the LU because of the current diet (2.03 m^2^*year/day), but safely within the planetary LU boundary of 2.68 m^2^*year/day (Table 5). In fact, LU for animal food products stays within the planetary boundary in all the scenarios.

**Table 5:** Overview of maximum nutrient intake recommendations exceeded (in italic) in the different scenarios.

**Scenario**	**Which boundary exceeded?**				
	**GHGE [kg CO_**2**_-eq/day]**	LU [m^2^*year/day]	**Daily choice [kcal/wk] (without butter)**	**Weekly choice [g/wk]**	**Explanation (main contributor(s) between brackets)**
FBDG boundary (36)			1,050	100	
Planetary boundary (4)	0.89	2.68			
Reference diet (10)	*3.53*	2.03			
1a maximum impact	*3.34*	2.44	*1,846* (42)	*142*	Daily (butter, beef cattle, broilers); Weekly (beef cattle, broilers).
1b realistic	*2.73*	1.62	*1,970* (90)	*154*	Daily (butter, pork, broilers); Weekly (pork, broilers, beef cattle).
2a minimum impact, near-vegetarian	***1.46***	**0.80**	*1,292* (4)	33	Daily (butter).
2b minimum impact, rooster meat	*1.47*	0.81	*1,292* (4)	35	Daily (butter).
3 food system optimal	*1.86*	1.15	*1,428* (84)	*122*	Daily (butter, pork, beef cattle); Weekly (pork, beef cattle, roosters).
4 agriculturally optimal	*2.79*	1.69	*1,965* (86)	*155*	Daily (butter, pork, broilers, beef cattle); Weekly (pork, beef cattle, broilers).
5 nutritionally optimal	*1.93*	1.20	1,048 (37)	100	Daily (butter, pork, broilers).

*Ecological impact per day (of the animal product consumption according to current Dutch FBDG) of the different scenarios vs. the current diet and planetary boundaries. Figures in bold are the lowest (4, 10)*.

The least greenhouse gas emissions (GHGEs) and land use (LU) within the Food-Based Dietary Guidelines (FBDG) can be achieved in a food system scenario without extra streams of meat (i.e., no beef cattle or broilers) and without roosters (Scenario 2a; minimum impact near-vegetarian scenario). Scenario 2b (minimum impact, white meat scenario) is nearly the same.

### Limiting Factors in Meat and Dairy Consumption

The results show that in all scenarios the category of Daily choice is a limiting factor, i.e., the caloric intake due to Daily choices exceeds 1,050 kcal/week (Table 5). The main contributor to the caloric intake of Daily choice is dairy fat (in the model as butter). By advising semi-skimmed milk, the dairy cow system produces for every daily portion of 450 ml of semi-skimmed milk (1.5% fat) 15.2 g of butter, and for every daily portion of 40 g of low-fat cheese (30% fat in dry matter; 18% fat) 21.2 g of butter. In total, 36.4 g of butter is more than six daily portions (Daily choice). The current situation is that most of this butter is exported, but if butter is to be consumed within the food system, it will be the food product that causes imbalance and excess kcal and saturated fat intake, and will be limited in the model. Indeed, further analysis excluding the caloric contribution of butter to Daily choice showed that Daily choice was far below the maximum recommendation of 1,050 kcal/week (Table 5; column 4 in parenthesis).

Besides the category of Daily choice, the category of Weekly choice is limiting in four of the scenarios, i.e., the amount of animal (-derived) products categorised as Weekly choice exceeds 100 g/week. The reason for exceeding the Weekly choice is primarily due to the consumption of pork, the most consumed meat, but the consumption of beef is also a contributor.

In contrast, the category of Weekly choice is not limiting in the following scenarios:

- in a situation in which no extra streams of meat exist (no beef cattle nor broilers) and no roosters are consumed (Scenario 2a minimum impact, near-vegetarian),
- in a situation in which no extra streams of meat exist but roosters are consumed (scenario 2b minimum impact, rooster meat), and
- in Scenario 5 (nutritionally optimal) in which Weekly choice is limited to 100 g/week.

### Livestock Size Impact

Dutch adults consume an average of 98 grammes of meat and meat products per day. Men eat more meat and meat products (115 g/day = 805 g/week) than women [81 g/day = 567 g/week; (27)]. Dutch adults prefer pork (47%), followed by chicken (29%), and beef (20%). Consumption of calf is very low: 1.7% (40). As the consumption of other animal species is <3%, it is excluded from this study. The Dutch self-sufficiency rates show that production and consumption in the country do not align (41). Especially, the veal industry produces substantially more than the Dutch population consumes, leading to a self-sufficiency rate for veal of 734%. All animal products have a self-sufficiency rate >100% (poultry 191%, eggs 314%, cheese 222%, butter 153%) except for (non-dairy herd) beef meat (59%). National beef cattle provide, on average, 17 g of beef *per capita* per week (25).

In all the scenarios, the recommended amounts of eggs, dairy, and cheese could be provided by a livestock size smaller than the current one, except for beef cattle in Scenario 1a (Table 6). Compared with current meat production numbers, consumption of animal (-derived) food products according to the FBDG in the current situation (i.e., in a food system without roosters but with beef cattle and broilers) requires annually just 51% of the dairy cows; 47% of the calves; 26% of the laying hens; and 26% of the broilers (Scenario 1a). In the food system optimal scenario (3), only a third of the cattle, a quarter of the laying hens, and a sixth of the pigs are needed. In the agriculturally optimal scenario (4), the livestock could be halved (except for beef cattle) to fulfil the FBDG-recommended amounts for the total population. In all the scenarios, a maximum of 14% of the current pig livestock is consumed.

**Table 6:** Estimated amounts of animals in the livestock production systems needed per year in the different scenarios, extrapolated to the Dutch population.

**Scenario**	**Dairy cattle**	**Calves**	**Beef cattle**	**Laying hens**	**Roosters**	**Broilers**	**Pigs**
1a	51%	47%	1,121%	39%	–	26%	–
1b	51%	47%	–	39%	–	27%	14%
2a	36%	33%	–	26%	–	–	–
2b	36%	33%	–	26%	29%	–	–
3	36%	33%	100%	26%	29%	–	14%
4	51%	47%	100%	39%	44%	26%	13%
5	29%	26%	100%	39%	44%	40%	3%
Production (100%)	522,300	1,629,800	71,500	17,951,700	= hens	605,487,800	15,907,000

*A hyphen means exclusion from the model in that specific scenario. Bottom row depicts current number of animals slaughtered for meat production in 2018 = 100% (25)*.

## Discussion

### The Food System Approach

This study explored the interconnection between food consumption and production in different food system scenarios. The main finding is that when taking interdependencies within the animal food system/livestock systems into account, it will affect the recommended intake of animal-based foods. Applying a more sustainable food system approach (i.e., eating the whole animal, utilising animal by-products, using waste streams) results in a decrease in GHGE, LU, and number of livestock compared with the current situation, but in some scenarios, it also increases the intake of SFAs, TFAs, and salt, and in most scenarios, it exceeded the recommended amount of Daily and/or Weekly choices. This approach asks for reconsideration of quantities of recommended cheese, total meat, and red meat, and for adaptations in consumer preferences for non-popular cuts, organ meat, veal, hens, roosters, and saturated fat source. The conceptual framework of this study may be useful in the discussion on how future sustainable FBDG can incorporate a more food system-based approach.

The main strength of this study is its novelty. Earlier research studied potential dietary changes, such as better adherence to healthy dietary patterns that could reduce the ecological impact of the diet, but not within a food system approach. For example, Vellinga et al. (10) evaluated the ecological impact of Dutch food consumption patterns by regression analysis and found that better adherence to Dutch healthy diet guidelines (35) for red and processed meat (less consumption) and vegetables (more consumption) was most strongly negatively associated with GHGE. This is in line with the findings of Van Dooren et al. (42) showing that eating a recommended healthy diet in compliance with the Dutch FBDG (2006) or other healthy diets such as the New Nordic and Mediterranean diet is likely to result in less GHGE and LU (42). This study shows for the first time that part of these conclusions (i.e., on red and processed meat) indeed also applies if the interconnections within the food system are considered.

Because of a lack of data, several assumptions were made, and several minor side streams were excluded, such as separator meats, unpopular organs such as intestines or hearts, and animal fat tissue. One of the arguable assumptions is that the ecological impact of all beef meat products is the same (beef and dairy cattle). Nevertheless, ecological impact data of beef and dairy cattle are only available on the level of the whole animal, and the differences are not to be neglected: the GHGEs and LU of beef cows are roughly four and five times higher than those of dairy cows, respectively (29). Also, only the highly productive Dutch livestock system was taken into consideration. Moreover, only few nutritional aspects were taken into consideration, without possible consequences for vitamin and mineral intake. Therefore, this study is more relevant for the conceptual framework including a novel food system approach rather than for its exact outcomes.

Nevertheless, several steps need to be taken to transform the current Dutch livestock production system and consumption of animal (-derived) products for all of the scenarios, even for the agriculture optimal one (4). In the end, many factors besides nutrition, including but not limited to politics, economics, health, ecology, and ethics together will shape the future food system. This study demonstrates only the impacts of possible scenarios from a public health and ecological food system approach.

### Economic Allocation of Ecological Impact

One of the most important and notable aspects in sustainable food research in general is that the current use of economic allocation is crucial, as it has an enormous effect on the ecological impact figures. Especially for by-products that have low ecological impacts according to this approach, i.e., roosters, laying hens, calves, animal fat, due to which the consumption of these products would theoretically be favourable from an environmental point of view (as in Scenario 3). Also, from a financial point of view, these by-products have the preference, as they come from waste or rest streams (i.e., pet food and feed) that currently are not used for human consumption. Such by-products, which are “free of charge” from an ecological impact perspective, disappear from the current, local human food system (i.e., these by-products end up in pet food and feed, or are exported). Meanwhile, part of the ecological impact has been made already in the country of origin. Therefore, from this perspective, using products, such as palm oil from abroad rather than local butter or animal fats, seems to be odd. This example demonstrates the ecological preference for animal by-products over additional imported ingredients with additional land use. Alternatives for economic allocation of ecological impact are physical allocation approaches such as mass or energy allocation (43). However, results in terms of GHGE and LU will inevitably be highly dependent on the choice of allocation (29, 43).

In the minimum scenario (2a, 2b), the amount of beef meat (dairy cow and calf) available in a diet is much more than the amount of chicken (laying hen) available. Contrary to this result, existing ecological impact data show that 1 kg of beef meat is less sustainable than 1 kg of chicken meat (28, 30). This contradiction can be explained by the fact that this study with a food system approach made use of existing ecological impact data that were not generated from a food system perspective.

### Considerations From the Nutrition Perspective

The focus of this study was to identify food products that would reduce the ecological impact without affecting health benefits substantially, to make optimal use of the interlinkage between animal (-derived) products between production and consumption. However, the health perspective should not be forgotten, as some by-products might be less healthier than the products it may substitute [for instance the high content of SFAs in butter vs. those in olive oil or sunflower oil (23)]. The results show that in some cases, using these by-products from rest streams with a minimum ecological impact does fit in a healthy dietary pattern, for instance organ and rooster meat that are included in the Wheel.

Another way to make optimal use of the interlinkages in the system of animal (-derived) products between production and consumption is to consume all safe parts of an animal rather than just the Wheel products. The results show that roughly a third (pork) to half (beef and chicken) of the hot carcass weight is classified as Wheel products. Consuming only the Wheel parts entails wasting a substantial part of the animal that is safe for human consumption. What is more, consumption of only Wheel parts requires a larger number of animals for the same amount of (Wheel) meat. In contrast, if we would consume all safe parts of the animals, food waste as well as the livestock size could be minimised (without taking export into account). An example are parts of pork that are nowadays very low in consumption (i.e., black pudding, cheeks, pork belly), most of which could be labelled Weekly choice. The drawback of such advice for the consumer is that the freedom to choose outside the Wheel will be limited.

Keeping the health perspective in mind, aligning production and consumption of animal products can also be stimulated when only Wheel products are taken into consideration (e.g., chicken fillet and chicken leg). However, the consumption of such Wheel products is not in line with the natural ratio of these components. According to a major chicken slaughterhouse, chicken fillet and chicken legs (i.e., drumsticks and chicken thighs) comprise about 24 and 48%, respectively, of the animal, giving a natural ratio fillet:legs of 1:2, whereas the current consumption of these parts is, respectively, 17.7 and 17.9 g/day, resulting in a “consumption ratio” fillet:legs of about 1:1. Consequently, in order to eat within the borders of the food system, either the consumption of chicken fillet should decrease by half or the consumption of chicken leg should double.

Based on the results, we calculated an extra Scenario 5 to keep the Daily and Weekly choices within the limits of the FBDG. This calculation demonstrates that cheese consumption and recommendation should be limited to about 20 g/day (145 g/week) and milk to the current lower recommendation of 300 mL/day. The maximum space for meat consumption in this scenario is 410 g of which a maximum of 140 g is for red meat. Both adjustments would also result in reduction of the environmental impact (GHGE −45%, LU −41%). However, we did not analyse the effects of these reductions on micronutrient intake yet, which is needed to check for sufficient intakes of, e.g., calcium, iron, and B-vitamins for Scenarios 2a, 2b, and 3. We recommend this additional analysis for future studies. The other scenarios fit completely within the guidelines. For nutrients that are mainly supplied by meat in the Dutch diet, the dietary reference level is not always achieved, both for Dutch vegetarian diets and for diets including meat. These are small differences with the standard, where the scenarios provide a level that is above or comparable with the current consumption (36). This is already taken into account through specific recommendations for meat-free diets within FBDG, e.g., it is recommended to consume foods naturally rich in iron, to use sufficient dairy and wholegrain products, and to consume meat replacements with sufficient protein and enriched with iron and thiamine or vitamin B12 (21). This is less urgent for the scenarios with a meat consumption of 84 to 500 g per week, although adequate replacement is recommended for all consumers.

Scenarios with a higher white meat consumption (1b, 4) demonstrate its positive effects on nutritional intake (SFAs, TFAs, salt) and GHGE, whereas scenarios with higher red meat consumption, such as high in pork (1b, 3, 4), demonstrate a negative effect on nutritional intake. Therefore, Scenario 5 (high white, low red meat) shows the best compromise for both health and sustainability, based on the indicators used in this study.

### Considerations From the Ecology Perspective

The results show that the Dutch FBDG regarding animal (-derived) products in the current situation can make steps forward to contribute to a more sustainable food system. The current GHGEs of the diet exceed the planetary boundary even if consumption is according to the lower limits of the FBDG. This is mainly because of beef cattle and dairy (especially cheese). In contrast, the FBDG do not exceed the planetary LU boundary. An explanation for this is that Dutch livestock production systems are relatively efficient (i.e., high milk and egg production per square metre) compared with those in other parts of the world, leading to an LU lower than the global limit. Moreover, GHGE exceeding the global boundary can be explained by the relatively large size of the Dutch animal husbandry (intensive use of inputs, fossil fuels, and infrastructure) and its inevitable emissions. Possible solutions are systems based on residue streams (17) or extensive farming of beef [grass-fed systems (44)].

Although the planetary LU boundary was not exceeded, the LU in Scenario 1a was calculated to be higher than the LU by the current reference diet. This might be explained by two assumptions of the model. First, the model assumed that butter was the only fats and oils product consumed in the FBDG, whereas the current Dutch population, in fact, consumes a mixture of animal and plant-based fats and oils, of which palm oil, for instance, has a lower LU than butter (32). Second, the model assumed a higher consumption of veal than currently consumed in the Netherlands, since most veal is exported (25). Existing data show that veal has a higher LU than dairy cows (28, 29). This study shows an evident gap between the production and consumption of veal. The previously mentioned 300 g of red meat available in Scenarios 1a, 1b, and 3 consists of almost a quarter of calf meat (67 g/week). In contrast, the current consumption of calves is only a tenth of this [1.7% of total meat (40)].

The current livestock size for meat production is substantially larger than necessary for a dietary pattern following the FBDG within a more optimal food system (Scenario 3): ca. three (calves and laying hens), to four (broilers), to six times (pigs) larger. This is in line with Dutch consumption data showing that the Dutch population consumes more meat in general than is advised by the FBDG (45). It is also in line with the fact that the Dutch agriculture is not focused on circularity, but on export (26). Interestingly, the results also show that the size of beef cattle would need to be five to six times larger than the current size in order to provide a diet with maximum of red meat as beef (Scenario 1a) within the FBDG. These extra beef cattle are not necessary in a scenario with more pork (1b). However, both scenarios underutilize the quantity of dairy cow beef available. The current dairy cow herd has this size, because there is a large export of cheese and other dairy products. Even in the agriculturally optimal Scenario 4, there is a significant reduction in livestock size for the internal market. The high rate of self-sufficiency at this moment (i.e., export dependency) is essential for the income of farmers. The stepwise transition to a more circular system with lower emissions, therefore, creates challenges to find other models of farming or sources of income.

Most of the non-meat portion of an animal carcass is biologically nutritious and can be made microbiologically safe for human consumption. However, because of individual preferences, the popularity of consumption of these products has decreased in contrast to the fact that meat consumption has increased (46). Edible by-products such as organs were widely used in culinary tradition in Europe, South America, North America, Asia, Africa, and Australia. In Africa, all parts of animals are still processed and commonly consumed as food for humans. A more efficient utilisation of animal by-products in the Netherlands and other western countries can alleviate the prevailing cost and scarcity of feed materials, which have high competition between animals and humans (47). This will also aid in reducing environmental pollution and increase the ecological efficiency of an animal-based food system.

The described dietary scenarios are more or less theoretical, including utilising old laying hens and non-popular parts of old dairy cows. The willingness of consumers to buy and pay for less than premium meat products remains to be seen.

### Limiting Factors in the Scenarios

Looking at the results in detail, we see three limiting factors in the scenarios: butter, beef cattle, and saturated fatty acids (SFAs).

- In all the scenarios, butter is the limiting factor by being the main contributor to the high caloric intake in the Daily choice category. Further analysis showed that lowering the recommendation of cheese from 40 to 20 g/day solved this problem. In the model, butter was chosen to be a Daily choice, because its caloric intake per portion of 6 g is only about half of the maximum caloric intake of one Daily choice (44 vs. 75 kcal), even though it does not fulfil the criteria for SFAs. Further research could, for example, investigate whether butter can replace other cooking fats and spreads that are based on plant-based fats with high SFA content and high environmental impact, i.e., palm fat and coconut fat (48).
- Pigs and beef cattle livestock systems are the main limiting factors concerning Weekly choice. From a food system perspective, dairy cattle and calves are given priority above beef cattle because of dairy production. Following this argumentation, beef cattle is redundant and even has a negative impact on nutritional intake through additional Weekly choices while having a diet according to the FBDG in the current situation. Whenever both beef cattle and broilers are absent, no nutritional limits (i.e., such as Weekly choice) are exceeded. Still, a limited quantity of beef cattle is possible from a food system perspective, no higher than the current production of beef cattle, and preferably from grass fed cattle, or cattle raised in nature sites (see scenario 3).
- A diet following the upper limits of the FBDG, regardless of whether extra meat streams are present, leads to a higher intake of SFAs and TFAs than the current intake from animal food products. One explanation is the substantial by-production of butter due to milk and cheese consumption, which is included in the calculation of nutritional intakes. Another explanation is the higher content of SFAs in the Weekly choices due to less lean and more processed meat. Another possibility is that the model in this study includes more room for Daily and Weekly choices, whereas the current FBDG contains amounts of Wheel products providing 85% of energy needs (36).

### Possible Steps to Make Guidelines More Sustainable

To move toward more sustainable livestock production systems and a balanced consumption of animal (-derived) food products, several steps can be taken to improve the current guidelines:

1. First, introducing the concept of consuming animals from nose to tail, and keeping the consumption of Weekly choice, SFAs, and TFAs within the FBDG limits would lead to lower maximum advice of red and total meat. This concept includes the promotion and consumption of less popular (lean) cuts and organs.
2. Second, recommending dairy and eggs from a health perspective in FBDG inevitably includes recommending a minimum consumption of meat on a population basis when adopting a food system approach. These quantities are low in a high productive food system. From this food system approach, vegetarian diets are less optimal than near-vegetarian diets. Considering waste streams, there would be some room for the consumption of pork (and broilers) fed on residue streams.
3. Third, introducing food system interdependencies includes the consumption of butter besides semi-skimmed milk and cheese. The limitations in SFAs, TFAs, and number of Daily choices, includes a limitation in butter and, therefore, dairy. A possible adaptation is to lower the recommendation of cheese, but cheese is also an important source of, e.g., calcium, and micronutrient intakes were not yet analysed. However, past optimisation studies from the group of the authors demonstrated that intakes of calcium and vitamins D, A, and B12 did not compromise lower quantities of milk and exclusion of cheese (42, 49).

These recommendations are in line with the ranges for animal products given by the EAT Lancet reference diet, but the averages are somewhat higher. This is because of the application to the local context of this study and the lack of a food system approach by Willett et al. (3).

Other sustainability scenario studies also suggest that within Dutch eating habits, satisfying optimisation constraints required a shift away from beef, cheese, butter, and snacks toward plant-based foods and fish and shellfish, questioning acceptability (50). A great deal of hope has been placed lately on a flexitarian diet to help solve food-related environmental sustainability problems. There is a growing group of flexitarians and vegetarians who would likely accept the described scenarios, but this is distinct from a substantial group of consumers who are deeply attached to meat-eating and have no intention whatsoever to limit their meat intake, let alone their already changing meat-eating behaviours (51). Other steps toward a more sustainable food system approach include minimising the production and consumption of beef cattle, pork, and broilers, choosing local animal (by-)products over foreign comparable products, maximising consumption of the whole animal, and exploring alternative applications of butter instead of plant-based saturated fats. Following these steps will help to prevent waste of rooster meat, organ meat, and other animal (by-)products considerably and would lower the number of livestock needed to fulfil a balanced, healthy consumption of animal (derived) products. The advice for future research is to also look at other food system interdependencies, for example, soy oil and soy pulp, fatty fish, and bycatch, grains, and legumes in agricultural rotations.

## Conclusions

This study provides a first insight into how the Dutch FBDG could be adapted to better align production and consumption of animal(-derived) food products within the Dutch food system, showing that the current FBDG could make steps toward a more sustainable food system when interdependencies in the animal production system are included. The major strengths of this study are its food system approach and its model, which can also be used to explore other food system scenarios. The main limitation is the lack of detailed LCA data generated with a food system approach. Hence, future studies on food systems need to be aware of the data gap on ecological impact data of different livestock types. Food systems-thinking involves shifts in the use of different livestock types in different livestock production systems. Addressing the data gap on differing livestock breeds is a first yet substantial subject for research to substantiate the change toward future sustainable food systems. Therefore, this study is more relevant for the conceptual framework and novel food system approach rather than for its exact outcomes. The conceptual framework of this study may be useful in the discussion on how future sustainable FBDG can incorporate a more food system-based approach, in addition to other preconditions applied to FBDG development, such as lower and upper level of nutrients, recommendations on food groups by the health council, and dietary habits of the population.

## Data Availability Statement

The Excel model with the data supporting the conclusions of this article will be made available by the authors on request, without undue reservation.

## Author Contributions

LM: methodology, investigation, formal analysis, and writing—original draft. CD: methodology, conceptualisation, formal analysis, writing—original draft, and visualisation. MS: writing—review & editing. SB: writing—review & editing and supervision. All authors contributed to the article and approved the submitted version.

## Conflict of Interest

The authors declare that the research was conducted in the absence of any commercial or financial relationships that could be construed as a potential conflict of interest.

## Publisher's Note

All claims expressed in this article are solely those of the authors and do not necessarily represent those of their affiliated organizations, or those of the publisher, the editors and the reviewers. Any product that may be evaluated in this article, or claim that may be made by its manufacturer, is not guaranteed or endorsed by the publisher.
